# Potential risk of brain damage and poor developmental outcomes in children prenatally exposed to SARS-CoV-2: a systematic review

**DOI:** 10.1590/1984-0462/2023/41/2021325

**Published:** 2023-03-13

**Authors:** Maria Tereza Rebello Corrêa, Vêronica Canarim de Menezes, Elonir Gomes, Elliane Mazzuco dos Santos

**Affiliations:** a Universidade do Sul de Santa Catarina, Tubarão, SC, Brazil.

Dear Editor,

It is with great pleasure that we read the study “‘Potential risk of brain damage and poor developmental outcomes in children prenatally exposed to SARS-CoV-2: a systematic review”, of great quantity and quality. We can see, even if in a limited way, the characteristics of the virus and its influence on the life of pregnant woman. For example, SARS-CoV-2 uses angiotensin-converting enzyme 2 (ACE2) as its receptor, contributing to the infection of human cells. Therefore, as ACE2 receptors are displayed in many tissues of the biological systems, including the brain, it may function as part of a biological mechanism that can lead to neurological complications.^1^

From this perspective, studies point to complications due to severe acute respiratory syndrome disease (SARS) and Middle East respiratory syndrome (MERS), which are associated with miscarriage, preterm delivery, intrauterine growth restriction, and maternal death.^2^ Therefore, it was not possible to find any studies on related abnormalities in the embryonic or fetal stages of brain development or to report a direct link between the virus and neurological abnormalities in human embryos, fetuses or children induced by SARS-CoV, MERS-CoV or SARS-CoV-2.^1^

Likewise, no evidence of in utero transmission was observed in SARS or MERS.^2^ However, there are studies showing that the presence of antibodies to SARS-CoV in breast milk can be influenced by the time of infection in relation to pregnancy.^3^ In addition, clinical and laboratory findings were similar to those seen in the non-pregnant population. Nonetheless, complications and adverse outcomes were more common among pregnant women: women who were pregnant had longer hospital stay, were statistically significantly more likely to develop renal failure, sepsis, and DIC, and were more likely to require intensive care unit admission.^2^

However, there are studies that prove that SARS-CoV can possibly infect multiorgan systems, and that the CNS could potentially be involved.^4^ Notwithstanding, there are still no studies directly proving the potential risk of brain damage and negative developmental outcomes of children prenatally exposed to SARS-CoV-2. In summary, an immediate conclusion cannot be drawn, as the discovery that the Zika virus is one of the factors for the increase of neurological damage in newborns in Maranhão^5^ is still recent, and was not accounted for at the time of the peak of the disease, the same can happen in cases with SARS-CoV-2.
